# Supplementary material for: T2DB: A Web Database for Long Non-Coding RNA Genes in Type II Diabetes
Source: Noncoding RNA. 2023 May 8;9(3):30. doi: 10.3390/ncrna9030030 (PMC10204529; doi:10.3390/ncrna9030030)
Supplement: Supplementary file 1 [file ncrna-2337344-supplementary figure proofreading back.pdf › ncrna-2337344-supplementary proofreading back/ncrna-2337344-supplementary figure proofreading back.pdf]

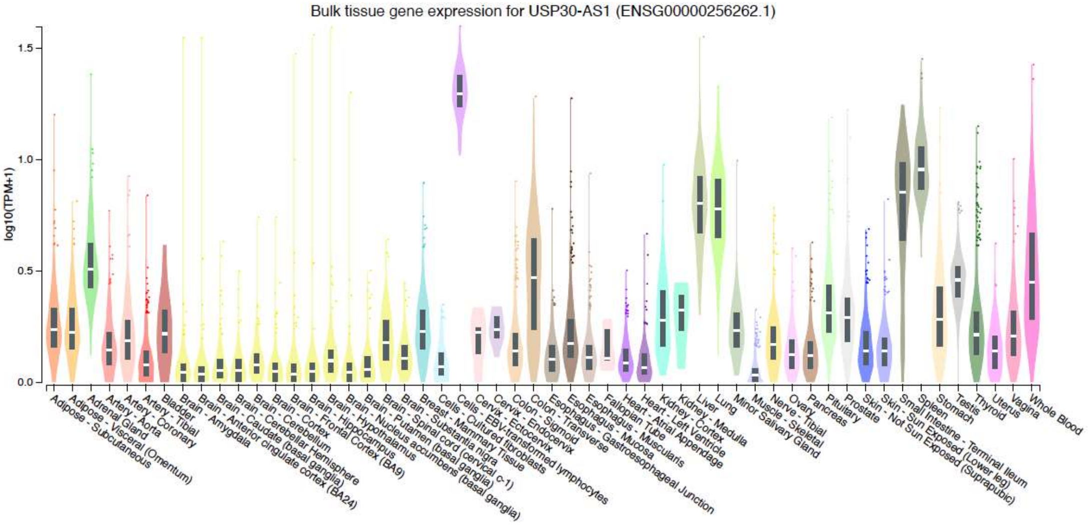

Supplementary Figure 1: Violin plots showing bulk tissue expression for the lncRNA gene *USP30-AS1*.
